# The Effect of Childhood Maltreatment on Affect Labeling in Youth: An ERP Study

**DOI:** 10.1002/brb3.70929

**Published:** 2025-10-07

**Authors:** Yuanyuan Li, Pengfei Yue, Mengmeng Shi

**Affiliations:** ^1^ College of Sports Medicine Wuhan Sports University Wuhan China; ^2^ Institute of Education Sciences Hubei Normal University Huangshi China; ^3^ Institute of Education Sciences Xinyang Normal University Xinyang China; ^4^ Faculty of Education Henan Normal University Xinxiang China

**Keywords:** affect labeling, childhood maltreatment, implicit, LPP

## Abstract

**Background:**

Previous studies on emotional processing in individuals with a history of childhood maltreatment have primarily examined either implicit or explicit processing modes in isolation, with limited research integrating both approaches. Empirical evidence regarding the use of affect labeling to regulate emotions among young adults with childhood maltreatment remains lacking. The affect labeling paradigm used in this study incorporates both implicit and explicit processing mechanisms, thereby offering a comprehensive means to investigate how childhood maltreatment specifically influences emotional processing.

**Method:**

This study employed event‐related potential (ERP) techniques to examine the influence of maltreatment on the affect labeling effect, using both affect labeling and gender labeling tasks. Participants were classified into maltreated (*n* = 17) and non‐maltreated (*n* = 19) groups based on their scores on the Childhood Trauma Questionnaire‐Short Form (CTQ‐SF).

**Results:**

(1) At Cz and CPz, the LPP amplitudes induced by affect labeling were significantly smaller than those induced by gender labeling. (2) At Fz, FCz, Cz, CPz, and Pz, maltreated individuals exhibited significantly lower LPP amplitudes compared to non‐maltreated individuals. (3) With respect to emotional categories, the LPP amplitudes in response to happy faces were smaller than those to angry faces.

**Conclusion:**

The findings suggest that implicit emotion regulation is beneficial for young adults with a history of childhood maltreatment in terms of emotion management. Maltreated individuals demonstrated decreased emotional arousal across both implicit and explicit emotional tasks, confirming the influence of maltreatment on the affect labeling effect. This supports the disruption theory. The present study expands the application of the affect labeling effect and provides new electrophysiological evidence for research on cognitive changes in individuals with childhood maltreatment.

## Graphical abstract

Utilizing ERP technology, this study investigated the impact of childhood maltreatment on affect labeling. Despite exhibiting generally lower levels of emotional arousal, young adults with a history of childhood maltreatment were found to employ affect labeling to regulate their emotions. These findings offer novel intervention strategies for addressing the adverse effects associated with childhood maltreatment.

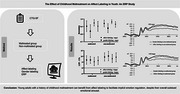

## 1 Introduction

Childhood maltreatment impacts the individual's emotional, behavioral, cognitive, and social functioning, persisting throughout their lifespan, which is defined as potential or substantial harm to the child's mental and physical health as a result of the child's essential requirements not being met (Koss 2019; Turgeon et al. 2020). Numerous studies have linked childhood maltreatment to a range of mental disorders, including post‐traumatic stress disorder, depression, bipolar disorder, and schizophrenia (Agnew‐Blais and Danese 2016; Liu et al. 2011; Norman et al. 2012; Yehuda et al. 2001), as well as to externalizing behaviors such as self‐harm and suicidality (Hein and Monk 2017; Heleniak et al. 2016). Importantly, individuals with a history of maltreatment often exhibit abnormalities in emotional processing and regulation even before the emergence of specific externalizing symptoms (Pears and Fisher 2005; Pollak et al. 2000).

Several studies on implicit emotion processing have revealed a robust association between maltreatment and attentional bias toward threatening information. For instance, maltreated toddlers tend to allocate greater early facial visual attention to angry faces (Curtis and Cicchetti 2011, 2013). Abused children also devote more attention resources to angry faces and have difficulty diverting their attention away from angry facial expressions (Pollak and Tolley‐Schell 2003). Young adults with moderate to severe child abuse exhibit a preference for paying attention to angry faces and are more sensitive in detecting angry expressions at lower emotional intensities (Gibb et al. 2009). In contrast, research on explicit emotion processing has shown that children of highly critical parents exhibit reduced LPP amplitudes when recognizing emotional faces, indicating diminished sustained attention to emotional stimuli across varying emotional intensities (James et al. 2018). Collectively, these findings suggest that childhood maltreatment disrupts both implicit and explicit emotional processing. Such impairments reflect broader deficits in the development of emotion regulation abilities.

Whether it is implicit or explicit emotional processing, it not only affects the initial emotional processing but also continuously influences the entire emotion regulation process. According to the process model of emotion regulation, emotional responses involve four stages: (a) Emotional responses are triggered by internal or external situations. (b) Attention is given to this situation. (c) The context relevant to the objectives is assessed. (d) An emotional reaction occurs (McRae and Gross 2020). The application of emotion regulation strategies might occur during any of the four phases. The impact of these strategies varies among individuals who have experienced abuse (Weissman et al. 2019). Individuals with maltreatment tend to utilize maladaptive emotion regulation strategies, including rumination and expression suppression (Weissman et al. 2019). For example, abuse has been linked to heightened rumination (Heleniak et al. 2016), while parenting behaviors like inconsistent discipline and physical punishment are associated with increased use of expressive suppression in children (Balan et al. 2017). Notably, one self‐report study found that abused adolescents did not differ from their non‐abused peers in their ability to use cognitive reappraisal; however, they exhibited greater prefrontal activation and expended more cognitive effort to attenuate negative emotions (McLaughlin et al. 2015). This suggests that although cognitive reappraisal, an adaptive emotion regulation strategy, remains accessible to this population, its practical application may require greater effort. Moreover, individuals with less severe abuse histories may employ cognitive reappraisal more effectively (Wooten et al. 2022). Specifically, acceptance‐based strategies appear to help adults with histories of childhood abuse mitigate negative emotions on an unconscious level, irrespective of abuse severity (Wooten et al. 2022). These findings underscore the potential benefits of investigating implicit emotion regulation, which requires minimal conscious effort, as a promising approach to supporting the mental well‐being of individuals affected by childhood maltreatment.

Affect labeling, which is a form of implicit emotion regulation through verbal encoding of emotional stimuli, functions without conscious supervision or explicit intent (Lieberman et al. 2011; Torre and Lieberman 2018). It involves using language, either spoken or written, to identify, describe, and articulate one's internal subjective affective states (Torre and Lieberman 2018). This process includes not only discrete emotions such as anger or fear but also broader affective experiences like stress and discomfort (Torre and Lieberman 2018; Yue et al. 2024). Studies show that affect labeling, such as expressive writing and journaling, can reduce test‐related anxiety in adolescents and help alleviate anxiety symptoms in parents (Morelen et al. 2013; Ramirez and Beilock 2011). Neuroimaging evidence from fMRI studies further supports these findings, indicating that expressive writing engages inhibitory mechanisms related to affect labeling (Memarian et al. 2017; Taylor et al. 2006). The inhibitory effect of affect labeling occurs through activation of the right ventral prefrontal cortex, which stimulates the fiber tracts in the mid‐prefrontal cortex and inhibits the activation state of the amygdala (Torre and Lieberman 2018). In high‐risk families characterized by rigor, confusion, and conflict, the neural connectivity of offspring to complete the affect labeling task is different from that of non‐risk offspring; that is, the activation of the ventrolateral prefrontal cortex in the offspring of high‐risk families is highly positively correlated with the activation of the amygdala (Taylor et al. 2006). In addition, a behavioral study of affect labeling reveals that decreased maternal accuracy in identifying children's facial emotions is associated with childhood physical abuse, emotional abuse, and physical neglect, whereas increased maternal accuracy in identifying anger is associated with emotional neglect (Turgeon et al. 2020). Young adults exposed to emotional neglect are slower at identifying emotional faces than controls (Jin et al. 2023). These findings imply that maltreated individuals may exhibit impaired emotion suppression during affect labeling.

Affect labeling effectively suppresses negative emotional responses, but it is important to note that this inhibitory effect arises only after the emotional naming process has been completed (Yue et al. 2015). In subsequent event‐related potential (ERP) studies on affect labeling, the late positive potential (LPP) has been widely adopted as an electrophysiological measure of an individual's emotional state following affect labeling. Studies have indicated that a decrease in LPP amplitude can be induced not only by labeling external emotional stimuli but also through directly naming one's own emotional states (Yue et al. 2024, 2016). Building on these earlier findings and experimental paradigms, the present study utilizes the LPP as its primary electrophysiological indicator. The LPP is a late component of ERPs, originating mainly from the parietal and occipital cortices. It typically emerges around 300 ms after stimulus onset and exhibits a sustained waveform that persists throughout stimulus presentation. Regarded as a reliable neural correlate of sustained attention to emotionally salient visual information (Moran et al. 2013; Thiruchselvam et al. 2011), the LPP shows enhanced amplitudes in response to emotionally salient compared to neutral stimuli (Hajcak and Olvet 2008; Lang and Bradley 2010). Its amplitude also varies systematically with emotional intensity, not only in threatening faces (Duval et al. 2013), but also across other emotional expressions (Olofsson et al. 2008). Due to these properties, the LPP serves as a neural marker of altered emotional processing following childhood abuse (McLean et al. 2020) and provides a sensitive metric for investigating the link between early maltreatment and sustained reactivity to ambiguous threats in adulthood (Sandre et al. 2018).

Most studies on emotion regulation in child maltreatment employ a single experimental task to assess either implicit or explicit emotional processing, resulting in a lack of ERP evidence that concurrently captures both modes of processing. Investigating implicit and explicit emotional processing in special populations can not only enhance our understanding of individual emotional development but also inform more effective emotional interventions. Previous studies have typically demonstrated the inhibitory effect of affect labeling through comparisons with the gender labeling task (Taylor et al. 2006; Yue et al. 2015, 2016, 2020). Therefore, participants were required to complete two emotional processing tasks: explicit (affect labeling) and implicit (gender labeling). The current study combined with ERPs to explore the effects of childhood maltreatment on the affect labeling effect of angry and happy faces. The hypotheses were presented as follows: (1) childhood maltreatment would block the affect labeling effect, meaning that LPP amplitudes of affect labeling in the maltreated group were not different from those of gender labeling; (2) cognitive processing was impaired in adults with childhood maltreatment, and the amplitudes of LPP in the maltreated group were lower than those in the non‐maltreated group.

## 2 Method

### 2.1 Participants

This study employed the Chinese version (Zhao et al. 2005) of the 28‐item Childhood Trauma Questionnaire‐Short Form (CTQ‐SF), a widely used retrospective instrument for assessing childhood maltreatment (Bernstein et al. 2003). The 28‐item questionnaire consists of 25 clinical items and 3 validity items and is organized into five subscales: emotional abuse, physical abuse, sexual abuse, emotional neglect, and physical neglect. An a priori power analysis conducted using G*Power software indicated that a total sample size of at least 24 participants was required to detect a medium effect size (*η_p_* ^2^ = 0.25) with 80% power (1 − *β* = 0.80) at a significance level of *α* = 0.05. A total of 725 questionnaires were randomly distributed to undergraduate students at a university in Central China. Participants were categorized based on their scores according to the classification criteria proposed by Bernstein et al. (2003) as adapted in the Chinese version (Zhao et al. 2005). Based on the top 15% of total questionnaires as the cutoff threshold, a subset of participants was randomly selected from this range, resulting in 17 individuals assigned to the high‐score group (maltreated group; CTQ‐SF scores range 53–76; nine males). Similarly, 19 participants were randomly selected from the lower end of the distribution to form the low‐score group (non‐maltreated group; CTQ‐SF scores range 25–26; six males).

All participants were between 18 and 25 years old (*M* = 20.00, SD = 1.27), with no history of neurological or psychiatric disorders. They had normal or corrected‐to‐normal vision, were right‐handed, and had no prior participation in any related behavioral or ERP experiments. Each participant received compensation upon completion of the study. The research protocol was approved by the Institutional Ethics Committee, and written informed consent was obtained from all participants prior to the experiment. All procedures were conducted in accordance with the principles of the Declaration of Helsinki.

### 2.2 Design

A mixed experimental design of 2 (group: maltreated group and non‐maltreated group) × 2 (emotion: happy and angry) × 2 (task: affect labeling and gender labeling) was adopted. Group served as the between‐subjects factor, while task and emotion were within‐subjects factors. The dependent variables were accuracy and LPP amplitude. Prior to the formal experiment, participants were asked to complete two scales: identifying and describing emotions in the alexithymia scale (Yi et al. 2003) and the positive affect and negative affect scale (PANAS) (Qiu et al. 2008). An independent samples *t*‐test was conducted using SPSS 26.0 to compare the two groups. Detailed information is presented in Table 1. No significant differences were found between the maltreated and non‐maltreated groups in age, emotion identification and description abilities, or current emotional state. However, a significant difference was observed between the two groups in terms of childhood maltreatment.

**TABLE 1:** Independent sample *t*‐test results of the two groups.

Variables	Maltreated group		Non‐maltreated group		*t*	df	95% CI Lower limit	95% CI Upper limit
	*M*	SD	*M*	SD				
Age	20.06	1.09	19.95	1.43	0.26	34	−0.76	0.98
CM	66.29	7.42	25.16	0.38	22.82***	34	37.32	44.96
IDE	32.18	5.32	30.95	7.59	0.56	34	−3.26	5.72
PANAS	69.94	7.23	72.53	6.97	−1.09	34	−7.40	2.23

Abbreviations: CI, confidence interval; CM, childhood maltreatment; IDE, identify and describe emotions; *M*, mean, SD, standard deviation.

*** *p* < 0.001.

### 2.3 Materials

A total of 120 pictures of emotional faces were selected from the Chinese Facial Emotional Picture System (CFAPS) (Gong et al. 2011), consisting of 60 happy and 60 angry faces with equal numbers of male and female models. The experimental materials were created using Flash. The experimental materials were developed using Flash. The screen background was set to white, with each facial image centered on the screen. The response prompt was a black “?” mark. Response options were displayed on the left and right sides of the “?” mark. The labels were evenly distributed between the two sides. In total, 120 pairs of affect‐labeling pictures and 120 pairs of gender‐labeling pictures were created.

The affect labeling condition was designated as group X, which was randomly and equally divided into subgroups X1 and X2. Similarly, the gender labeling condition was divided equally into two subgroups: Y1 and Y2. The entire experiment was structured into two distinct orders: X1–Y2–Y1–X2 and Y1–X2–X1–Y2. The two orders were counterbalanced across participants. A rest screen was included between each experimental block.

### 2.4 Procedures

The researchers explained the experiment's purpose and procedure to all participants in advance. The study took place in a soundproof, electromagnetically shielded room. Participants sat approximately 100 cm away from the computer screen. They were instructed to remain quiet and avoid unnecessary movements throughout the session. During the break, participants could rest with their eyes closed and were free to control the duration of their rest. The entire experiment lasted approximately 30 min. The experiment began with a practice phase, which included 16 trials of affect labeling and 16 trials of gender labeling. Participants proceeded to the formal experiment only if their practice accuracy exceeded 85%. Consistent with methodological standards in ERP studies on affect labeling, this accuracy threshold was implemented to ensure that participants reached a predefined performance criterion before advancing to the main trials. This approach enhances the validity of neural data by reducing variability due to inadequate task comprehension or low engagement, thereby improving the signal‐to‐noise ratio in ERP recordings (Yue et al. 2015, 2016, 2020).

**FIGURE 1:**
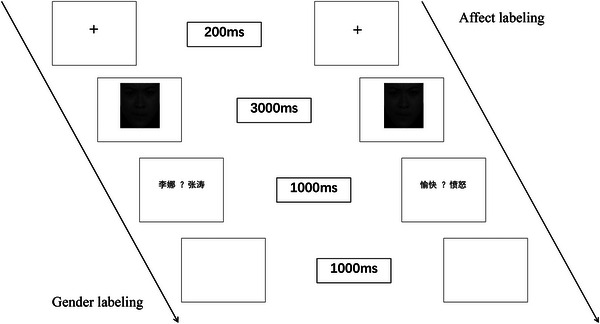
Example trial sequences in the labeling task.

The experimental procedure was as follows: First, a fixation cross (“+”) was presented for 200 ms. Then, a facial image was displayed for a fixed duration of 3000 ms. Subsequently, during the response interface, participants were required to judge either the gender or the emotion of the face previously shown (e.g., “张涛” for male, “李娜” for female). The response screen displayed two corresponding labels: one assigned to the left mouse button and the other to the right mouse button. The interface disappeared immediately after a button press. If no response was made, it disappeared after 1000 ms. Finally, a blank screen was presented for 1000 ms, after which the next trial began (see Figure 1).

### 2.5 Data Collection and Analysis

The experiment was programmed using E‐Prime 2.0, which also recorded behavioral data such as participant accuracy rates. We recorded the EEG using the NeuroScan SynAmp2 amplifier and a 64‐electrode cap. The left mastoid served as the reference electrode for collecting signal. Horizontal electrooculogram (HEOG) electrodes were placed approximately 1–2 cm outside the outer canthus of each eye, and vertical electrooculogram (VEOG) electrodes were attached above and below the left eye. The amplifier applied a bandpass filter of 0.05–100 Hz and a sampling rate of 1000 Hz. All electrode impedances were maintained below 5 kΩ.

Offline analysis was performed using Scan 4.5. The original reference electrode was re‐referenced to the average of the bilateral mastoids. Ocular artifacts were removed based on VEOG recordings during EEG preview. EEG epochs were extracted from −200 to 1000 ms relative to stimulus onset, with the 200 ms pre‐stimulus interval serving as the baseline. Segments exceeding ±100 µV after baseline correction were excluded. The remaining data were then averaged across trials and filtered with a 30 Hz low‐pass cutoff (24 dB/octave). Finally, the preprocessed data were subjected to ANOVA in SPSS 26.0, with *p* values corrected using the Greenhouse–Geisser method. The analysis included the following electrode sites: Fz, FCz, Cz, CPz, and Pz (Bai and Yue 2013; Yue et al. 2015).

## 3 Results

### 3.1 Behavioral Results

In accordance with established conventions (Cohen 1998), partial eta‐squared (*η_p_* ^2^) values of approximately 0.01, 0.06, and 0.14 were interpreted as indicating small, medium, and large effect sizes, respectively. The corresponding effect size is reported alongside each statistically significant result.

**FIGURE 2:**
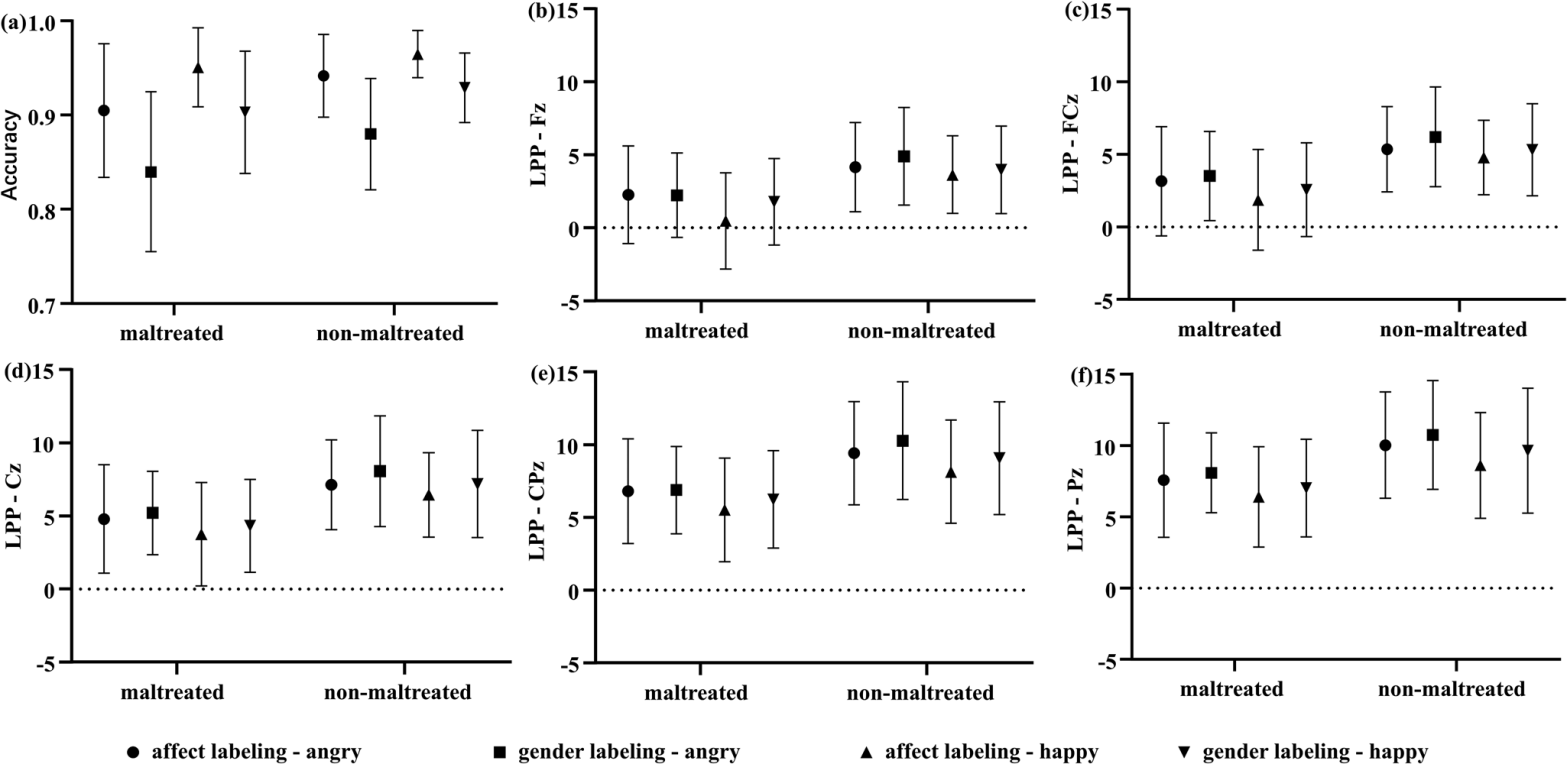
Results of repeated‐measures analyses of variance (ANOVAs) for behavioral and electrophysiological measures. (a) Behavioral accuracy across conditions. (b–f) Late positive potential (LPP) amplitude across conditions for the five electrode sites (Fz, FCz, Cz, CPz, Pz), respectively.

The repeated‐measures ANOVA on accuracy (see Table 2 and Figure 2 ) revealed a significant main effect of task, *F*_(1, 34)_ = 39.947, *p* < 0.001, *η_p_* ^2^ = 0.540 (large effect), with affect labeling accuracy being significantly higher than gender labeling accuracy. The main effect of group was also significant, *F*_(1, 34)_ = 4.158, *p* = 0.049, *η_p_* ^2^ = 0.109 (medium effect), indicating lower accuracy in the maltreated group. Similarly, the main effect of emotion was significant, *F*_(1, 34)_ = 60.429, *p* < 0.001, *η_p_* ^2^ = 0.640 (large effect), with higher accuracy for happy faces compared to angry faces.

**TABLE 2:** The accuracy in the maltreated and non‐maltreated groups.

Accuracy	Affect labeling				Gender labeling			
	Angry		Happy		Angry		Happy	
	*M*	SD	*M*	SD	*M*	SD	*M*	SD
Maltreated group	0.91	0.07	0.95	0.04	0.84	0.09	0.90	0.07
Non‐maltreated group	0.94	0.04	0.97	0.03	0.88	0.06	0.93	0.04

**FIGURE 3:**
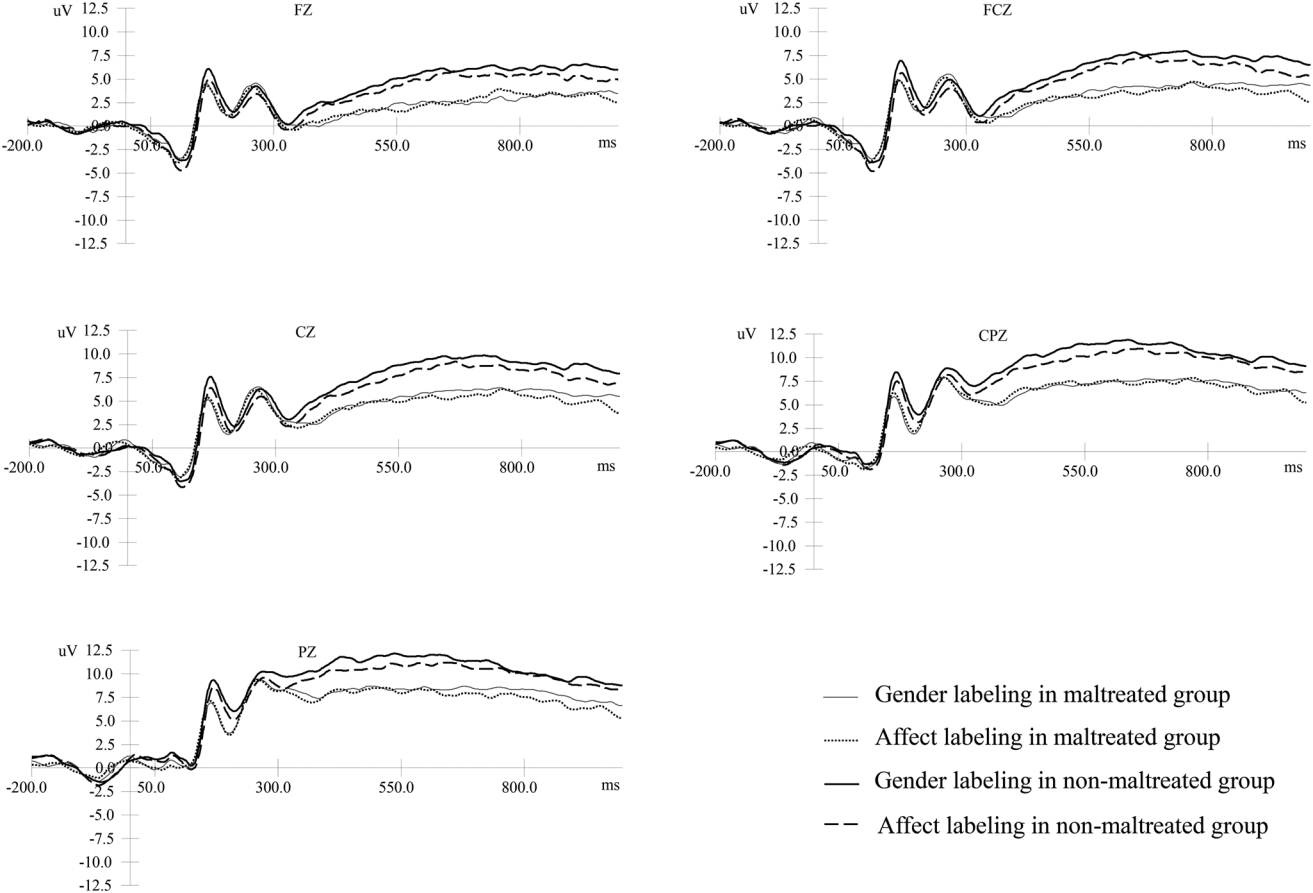
Grand average ERPs in response to angry faces.

None of the interactions were statistically significant: the group × emotion interaction, *F*_(1, 34)_ = 2.358, *p* = 0.134, *η_p_* ^2^ = 0.065; the group × task interaction, *F*_(1, 34)_ = 0.233, *p* = 0.632, *η_p_* ^2^ = 0.007; the emotion × task interaction, *F*_(1, 34)_ = 3.138, *p* = 0.085, *η_p_* ^2^ = 0.085; and the three‐way group × emotion × task interaction, *F*_(1, 34)_ = 0.139, *p* = 0.712, *η_p_* ^2^ = 0.004.

### 3.2 ERP Results

The results of the repeated‐measures ANOVA for LPP amplitudes across the five electrode sites (see Table 3, Figures 2, 3, 4) indicated that only the main effects were significant. The details are presented below:

1. Fz. The main effect of group was significant, *F*_(1, 34)_ = 7.658, *p* = 0.009, *η_p_* ^2^ = 0.184 (large effect), with the maltreated group showing lower average amplitude than the non‐maltreated group. The main effect of emotion was also significant, *F*_(1, 34)_ = 10.988, *p* = 0.002, *η_p_* ^2^ = 0.244 (large effect), with lower amplitudes in response to happy compared to angry faces. The main effect of the task was not significant, *F*_(1, 34)_ = 3.838, *p* = 0.075, *η_p_* ^2^ = 0.090.
  **TABLE 3:** The LPP amplitudes in the maltreated and non‐maltreated groups.

LPP		Affect labeling				Gender labeling			
		Angry		Happy		Angry		Happy	
		*M*	SD	*M*	SD	*M*	SD	*M*	SD
Fz	Maltreated group	2.25	3.34	0.48	3.30	2.23	2.90	1.78	2.96
	Non‐maltreated group	4.15	3.06	3.63	2.66	4.90	3.35	3.97	3.01
FCz	Maltreated group	3.15	3.77	1.87	3.46	3.52	3.08	2.58	3.24
	Non‐maltreated group	5.37	2.95	4.79	2.56	6.21	3.44	5.32	3.16
Cz	Maltreated group	4.80	3.71	3.77	3.53	5.22	2.86	4.34	3.17
	Non‐maltreated group	7.14	3.07	6.45	2.89	8.07	3.79	7.20	3.68
CPz	Maltreated group	6.81	3.60	5.53	3.57	6.89	3.01	6.24	3.35
	Non‐maltreated group	9.42	3.55	8.14	3.55	10.28	4.04	9.08	3.87
Pz	Maltreated group	7.59	4.01	6.42	3.52	8.11	2.80	7.04	3.43
	Non‐maltreated group	10.04	3.71	8.62	3.70	10.75	3.81	9.65	4.37
  **FIGURE 4:**
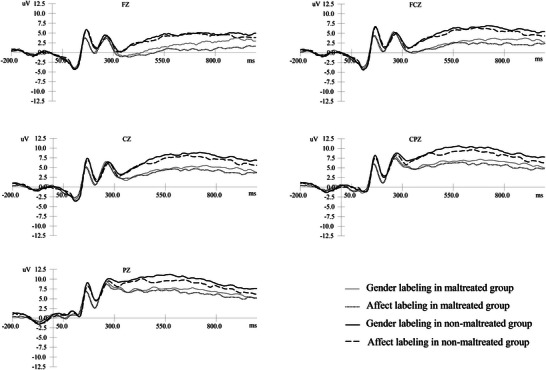
Grand average ERPs in response to happy faces.
  None of the interactions reached significance: group × emotion, *F*_(1, 34)_ = 0.487, *p* = 0.490, *η_p_* ^2^ = 0.014; group × task, *F*_(1, 34)_ = 0.023, *p* = 0.879, *η_p_* ^2^ = 0.001; emotion × task, *F*_(1, 34)_ = 0.690, *p* = 0.412, *η_p_* ^2^ = 0.020; and the three‐way group × emotion × task interaction, *F*_(1, 34)_ = 2.473, *p* = 0.125, *η_p_* ^2^ = 0.068.
2. FCz. The main effect of group was significant, *F*_(1, 34)_ = 7.753, *p* = 0.009, *η_p_* ^2^ = 0.186 (large effect), with the maltreated group exhibiting lower amplitudes than the non‐maltreated group. The main effect of emotion was also significant, *F*_(1, 34)_ = 8.708, *p* = 0.006, *η_p_* ^2^ = 0.204 (large effect), showing reduced amplitudes in response to happy compared to angry faces. The main effect of the task was not significant, *F*_(1, 34)_ = 4.115, *p* = 0.050, *η_p_* ^2^ = 0.108.
  None of the interactions reached statistical significance: group × emotion, *F*_(1, 34)_ = 0.370, *p* = 0.547, *η_p_* ^2^ = 0.011; group × task, *F*_(1, 34)_ = 0.066, *p* = 0.799, *η_p_* ^2^ = 0.002; emotion × task, *F*_(1, 34)_ < 0.001, *p* = 0.985, *η_p_* ^2^ < 0.001; and the three‐way group × emotion × task interaction, *F*_(1, 34)_ = 0.423, *p* = 0.520, *η_p_* ^2^ = 0.012.
3. Cz. The main effect of group was significant, *F*_(1, 34)_ = 7.187, *p* = 0.011, *η_p_* ^2^ = 0.174 (large effect), with the maltreated group showing lower amplitudes than the non‐maltreated group. The main effect of emotion was also significant, *F*_(1, 34)_ = 7.178, *p* = 0.011, *η_p_* ^2^ = 0.174 (large effect), indicating lower amplitudes in response to happy compared to angry faces. Additionally, the main effect of the task was significant, *F*_(1, 34)_ = 5.119, *p* = 0.030, *η_p_* ^2^ = 0.131 (medium effect), with affect labeling eliciting lower amplitudes than gender labeling.
  None of the interactions reached statistical significance: group × emotion, *F*_(1, 34)_ = 0.073, *p* = 0.788, *η_p_* ^2^ = 0.002; group × task, *F*_(1, 34)_ = 0.338, *p* = 0.565, *η_p_* ^2^ = 0.010; emotion × task, *F*_(1, 34)_ = 0.001, *p* = 0.972, *η_p_* ^2^ < 0.001; and the three‐way group × emotion × task interaction, *F*_(1, 34)_ = 0.118, *p* = 0.733, *η_p_* ^2^ = 0.003.
4. CPz. The main effect of group was significant, *F*_(1, 34)_ = 6.955, *p* = 0.013, *η_p_* ^2^ = 0.170 (large effect), with the maltreated group exhibiting lower amplitudes than the non‐maltreated group. The main effect of emotion was also significant, *F*_(1, 34)_ = 13.039, *p* < 0.001, *η_p_* ^2^ = 0.277 (large effect), showing reduced amplitudes in response to happy compared to angry faces. Additionally, the main effect of the task was significant, *F*_(1, 34)_ = 4.335, *p* = 0.045, *η_p_* ^2^ = 0.113 (medium effect), indicating lower amplitudes during affect labeling than during gender labeling.
  None of the interactions reached statistical significance: group × emotion, *F*_(1, 34)_ = 0.216, *p* = 0.645, *η_p_* ^2^ = 0.006; group × task, *F*_(1, 34)_ = 0.657, *p* = 0.423, *η_p_* ^2^ = 0.019; emotion × task, *F*_(1, 34)_ = 0.497, *p* = 0.486, *η_p_* ^2^ = 0.014; and the three‐way group × emotion × task interaction, *F*_(1, 34)_ = 0.281, *p* = 0.599, *η_p_* ^2^ = 0.008.
5. Pz. The main effect of group was significant, *F*_(1, 34)_ = 5.059, *p* = 0.031, *η_p_* ^2^ = 0.130 (medium effect), with the maltreated group exhibiting lower amplitudes than the non‐maltreated group. The main effect of emotion was also significant, *F*_(1, 34)_ = 14.044, *p* < 0.001, *η_p_* ^2^ = 0.292 (large effect), indicating reduced amplitudes in response to happy compared to angry faces. The main effect of the task was not significant, *F*_(1, 34)_ = 3.572, *p* = 0.067, *η_p_* ^2^ = 0.095.

None of the interactions reached statistical significance: group × emotion, *F*_(1, 34)_ = 0.046, *p* = 0.832, *η_p_* ^2^ = 0.001; group × task, *F*_(1, 34)_ = 0.153, *p* = 0.698, *η_p_* ^2^ = 0.004; emotion × task, *F*_(1, 34)_ = 0.145, *p* = 0.706, *η_p_* ^2^ = 0.004; and the three‐way group × emotion × task interaction, *F*_(1, 34)_ = 0.043, *p* = 0.836, *η_p_* ^2^ = 0.001.

## 4 Discussion

There is still a lack of empirical evidence on whether young adults exposed to childhood maltreatment are able to use affect labeling to regulate their emotions. In the present study, we employed both affect labeling and gender labeling tasks to examine how maltreatment influences the affect labeling effect in young adults. Our findings indicate that affect labeling, as an implicit regulatory strategy, may support emotion regulation among young adults who have experienced childhood maltreatment. Furthermore, consistently low arousal levels across implicit and explicit emotional processing tasks suggest that maltreated individuals exhibit impairments in emotional processing.

The LPP amplitudes during affect labeling were lower than those during gender labeling, indicating that emotions evoked by facial expressions were suppressed following affect labeling. This demonstrates that the affect labeling effect can also occur among individuals with a history of maltreatment. The results fail to support Hypothesis 1 and are inconsistent with previous studies on offspring from high‐conflict families (Taylor et al. 2006). A possible explanation for this discrepancy may lie in the observed suppression of the LPP, which suggests that the neural circuitry supporting affect labeling could remain functionally intact in maltreated individuals. The inhibitory neural networks involved in affect labeling may maintain functional connectivity in these individuals. Once an emotion is verbally labeled, the affect labeling process can still effectively generate inhibitory regulation. Furthermore, differences in participant characteristics between the two studies, such as age, type of maltreatment, and severity of abuse, may also contribute to the divergent findings. Specifically, in Taylor et al. (2006), participants ranged from 18 to 35 years old, and family conflict was characterized by emotional abuse, minor physical abuse, and exposure to interparental violence. In the current study, the average age of participants was 20 years, and scores on trauma questionnaires were significantly higher. Nevertheless, the affect labeling effect observed in both groups supports the disruption theory, which asserts that when individuals process negative emotions consciously, the unconscious induction of the emotion will be inhibited (Torre and Lieberman 2018; Yue et al. 2015). Previous studies on affect labeling have shown that the LPP amplitudes of healthy subjects are much lower during affect labeling than during gender labeling (Bai and Yue 2013; Yue et al. 2015). Meanwhile, the affect labeling effect is conducive to reducing negative emotions in individuals with post‐traumatic stress disorder, autism, and arachnophobia (Torre and Lieberman 2018). In conclusion, affect labeling helps adults exposed to childhood maltreatment regulate their emotions implicitly.

The LPP amplitudes at the Fz, FCz, Cz, CPz, and Pz electrode sites were significantly lower in the maltreated group than in the non‐maltreated group, validating Hypothesis 2 and indicating that the maltreated group allocates fewer attentional resources in the later stages of facial processing. Teenagers from high‐conflict families are more likely to exhibit an escape/avoidance response (Valentiner et al. 1994). Similarly, offspring from high‐risk families show lower levels of amygdala activation and a tendency to avoid stimuli when passively viewing angry or fearful faces (Taylor et al. 2006). Young children exposed to environmental violence and those who were frequently criticized by their parents also demonstrated lower LPP amplitudes in response to angry faces, indicating a cognitive avoidance pattern during the later stages of processing negative facial stimuli (Goldstein et al. 2021; James et al. 2018). The vigilance‐avoidance theory offers a plausible explanation for this pattern. This theory proposes that anxious individuals exhibit a specific pattern of cognitive resource deployment when confronted with threat‐related stimuli: resources are initially allocated to the stimuli, but cognitive avoidance occurs in the later stages, involving comprehension, attention, and memory (Derakshan et al. 2007). Reduced sustained attention to threatening information in the later stages may serve to attenuate negative emotional experiences. Thus, maltreated individuals may have learned to shift attention away from stressors as a means of coping with persistent or uncontrollable threats (Hoepfel et al. 2022).

The amplitudes of the LPP in the maltreated group were also lower than those in the non‐maltreated group during the happy face labeling task. This may be caused by impaired emotional discrimination in the maltreated group (van Harmelen et al. 2013), which is consistent with their behavioral performance. Additionally, a hostile attribution bias in the maltreated group might also play a role. Individuals with a history of maltreatment tend to attend to hostile cues (Dodge 1993). Consequently, smiles might be perceived as derisive or malicious, judged as threatening stimuli (Pine et al. 2005), and ultimately elicit an avoidance pattern similar to that seen with angry faces.

The LPP amplitudes in response to angry faces were larger than those in response to happy faces, indicating that individuals allocate more cognitive resources to processing angry faces during the later stages of emotional processing. This result is consistent with previous studies and aligns with the principles of evolutionary psychology (Calvo and Beltrán 2013; Yuan et al. 2012). Evolutionary psychology posits that maintaining appropriate vigilance toward threats is essential for individual survival and development, as it helps avoid potential dangers and injuries in the environment (Lo and Cheng 2017).

The current study provides electrophysiological evidence for neural alterations in the processing of emotional facial expressions in youth with a history of childhood maltreatment. Affect labeling, particularly as a low‐cost and nonintrusive technique, can be incorporated into therapeutic interventions for individuals with childhood trauma. We specifically propose that it may serve as an initial emotion regulation skill‐building module within broader trauma‐focused therapies, such as dialectical behavior therapy or trauma‐focused CBT. This approach could help individuals alleviate distress and enhance their engagement in subsequent treatment. Several limitations warrant mention. The use of retrospective self‐reports may introduce recall bias, and the gender‐imbalanced sample limits its generalizability. Furthermore, the study did not differentiate between maltreatment subtypes. Reaction times were not collected during facial stimulus presentation, although this design choice prioritized ERP signal quality. Finally, the exclusive use of Chinese stimuli constrains cross‐cultural applicability. Future studies should incorporate multimodal assessment, larger and balanced samples, subtype differentiation, comprehensive behavioral data, and culturally diverse stimuli.

## 5 Conclusion

As an implicit emotion regulation strategy, affect labeling can help young adults with childhood maltreatment regulate their emotions and maintain their mental health. Maltreated young adults showed lower emotional arousal in both implicit and explicit emotional tasks, indicating that childhood maltreatment hinders emotional processing and has long‐term adverse effects on cognitive processing.

## Author Contributions

**Yuanyuan Li**: conceptualization, methodology, data curation, investigation, writing – original draft, writing–review and editing. **Pengfei Yue**: conceptualization, writing – review and editing. **Mengmeng Shi**: writing – review and editing.

## Ethics Statement

All procedures involving human participants were approved by the institutional and/or national research committee and were performed in accordance with its ethical standards. This study was also guided by the principles of the 1964 Helsinki Declaration and its later amendments.

## Consent

Written informed consent was obtained from all participants prior to the study's commencement. Participants were informed that their data would be published and provided consent accordingly.

## Conflicts of Interest

The authors declare no conflicts of interest.

## Peer Review

The peer review history for this article is available at https://publons.com/publon/10.1002/brb3.70929.

## Data Availability

The data supporting this study's findings are available from the corresponding author upon reasonable request. However, they are not publicly available due to privacy and ethical restrictions.
